# Bleaching Performance and Mechanism of Al-MCM-41 Tuned by Si/Al in Rapeseed Oil

**DOI:** 10.3390/foods15101738

**Published:** 2026-05-14

**Authors:** Yu Wang, Chengming Wang, Guowei Ling, Mingshuang Xia, Yuhan Yi, Shilin Liu, Wenlin Li

**Affiliations:** 1 College of Food Science and Technology, Huazhong Agricultural University, Wuhan 430070, China; wy0912@webmail.hzau.edu.cn (Y.W.);; 2 Key Laboratory of Environment Correlative Dietology, Huazhong Agricultural University, Ministry of Education, Wuhan 430070, China; 3 Oil Crops and Lipids Process Technology National & Local Joint Engineering Laboratory, Oil Crops Research Institute of the Chinese Academy of Agricultural Sciences, Wuhan 430062, China

**Keywords:** Al-MCM-41, bleaching, mechanism, rapeseed oil, Si/Al ratio, regenerable adsorbent

## Abstract

Traditional activated clay (AC) bleaching usually shows limited adsorption selectivity, leading to micronutrient loss during pigment removal, and also suffers from high residual oil retention and poor regenerability. Developing mild bleaching materials with both high adsorption efficiency and selectivity is therefore important for oil refining. Mesoporous Al-MCM-41 (AM) adsorbents with different Si/Al ratios were prepared and characterized in pore structure and acidity, and the bleaching performance against AC in terms of pigment removal and the retention of micronutrients in rapeseed oil and the bleaching mechanism were studied. The results showed that AM25 (Si/Al = 25) exhibited the best overall performance among the AM samples under the tested conditions (70 °C, 20 min). It achieved a bleaching efficiency of 92.3% and removed 94.56% of chlorophyll, 92.94% of lutein, and 84.09% of β-carotene. In addition, AM25 reduced the peroxide value from 2.52 to 0.58 mmol/kg. High retentions of tocopherols (93.89%), phytosterols (98.73%), and squalene (96.32%) were also observed. Meanwhile, the adsorption rates of α-tocopherol, brassicasterol, and α-linolenic acid showed the highest values in their relative homologues of tocopherols, phytosterols, and free fatty acids (FFAs), respectively, due to differences in the methyl amount of tocopherols, the side-chain unsaturation of phytosterols, and the fatty acid chain unsaturation of fatty acids. Furthermore, the kinetic and isotherm data for chlorophyll and carotenoids were better described by the pseudo-second-order and Freundlich models, respectively. Combined with thermodynamic analysis, they indicated that adsorption was a spontaneous, endothermic, entropy-driven, heterogeneous multilayer process dominated by physical adsorption. Further, pigment adsorption was mainly governed by uniform mesopores and Si–OH/Si–OH–Al sites in AM. Among them, carotenoid removal depended primarily on the dispersion effect of moderately strong acid sites within pore-confined regions, whereas chlorophyll removal was more sensitive to the number of acidic sites in AM. AM25 still maintained 83.31% bleaching efficiency after five regeneration cycles. These performances of AM25 are significantly superior to that of AC.

## 1. Introduction

Crude vegetable oils typically contain natural pigments such as chlorophylls and carotenoids, which collectively determine the initial color and storage stability of oils [1,2]. The color of vegetable oil has a significant impact on the visual presentation of dishes or food products, serving as an immediate indicator of quality for consumers [3]. Consequently, refining and bleaching processes are of paramount importance in achieving a consistent appearance and product uniformity.

Among these pigments, chlorophyll and its derivatives act as photosensitizers, promoting the generation of singlet oxygen (^1^O_2_), which accelerates lipid oxidation and reduces oil stability [4]. Therefore, reducing chlorophyll content is essential to mitigate photo-oxidation during storage and transportation. In contrast, carotenoids contribute to the color of oils from yellow to orange-red and exhibit anti-oxidant activity, while β-carotene in carotenoids also serve as provitamin A compounds [5]. However, they may shift from anti-oxidant to pro-oxidant behavior under high-temperature conditions and high concentration conditions [6]. In addition, for high-temperature applications such as frying, lighter-colored refined oils generally exhibit lower free fatty acid (FFA) content and higher smoke points, making the oils more suitable for thermal processing [7,8]. Moreover, pigments significantly influence the appearance consistency of end products such as mayonnaise, emulsified sauces, and bakery fats [9,10,11]. In addition to pigments, vegetable oils are rich in micronutrients such as tocopherols, phytosterols, and squalene, which play crucial roles in the oxidative stability and nutritional functionality of oils. Among them, tocopherols protect polyunsaturated fatty acids in cell membranes and lipoproteins against oxidative damage [12], phytosterols reduce LDL-C through competitive inhibition of cholesterol absorption [13], and squalene, as a sterol precursor, contributes to the anti-oxidant defense of the skin barrier [14].

At present, adsorption bleaching is the predominant bleaching approach in vegetable oil refining, and AC is widely used in industrial bleaching due to its low cost, availability, and enabling of the simultaneous removal of pigments and certain polar impurities under vacuum conditions [15]. However, several limitations have become increasingly evident. First, the deficiency of strong adsorption selectivity leads to significant losses of lipid-soluble micronutrients, with tocopherol losses during bleaching commonly reported at about 11–27%, whereas phytosterol losses are generally lower, around 2–10% [16,17,18]. Second, the process typically requires higher temperatures (90–120 °C), which increases energy consumption and promotes the oxidation and degradation of heat-sensitive components such as tocopherols, phytosterols, and polyphenols [1]. Furthermore, AC contains residual oil and poses risks of spontaneous combustion, and its disposal mainly relies on landfilling, leading to environmental concerns [19]. Therefore, the development of novel adsorbents with strong adsorption selectivity at lower temperatures while maintaining reusability has become a critical research focus.

In recent years, various emerging adsorbents have been reported for vegetable oil bleaching. Metal–organic frameworks, such as UiO-66-COOH/chitosan aerogels and MIL-88B (Fe), have demonstrated potential for pigment removal, with adsorption behavior evaluated through kinetic and isotherm models [20,21]. Mesoporous silica materials functionalized with amine groups and different pore sizes were employed in a crude olive oil system, showing that small mesopores of approximately 5 nm were more effective in achieving the synergistic removal of free fatty acids and chlorophyll. Similarly, mesoporous silica aerogels derived from rice husk ash were applied to sunflower oil refining at 90 °C for 30 min, where aerogels exhibited superior multi-component purification performance, achieving up to 32.2% FFA removal at a dosage of 3 wt% while simultaneously reducing color pigments and phosphorus [22]. In addition, thermally reduced graphene oxide was used for bleaching neutralized rapeseed oil and achieved 91.7% chlorophyll removal at 30 °C within 10 min while largely retaining endogenous micronutrients [23]. However, these studies have still focused mainly on the adsorption performance of the adsorbents, whereas the roles of pore structure and acidity in regulating pigment removal and the selective adsorption or retention of minor components, including micronutrients and FFAs, remain insufficiently clarified. This knowledge gap limits the rational design of selective and regenerable adsorbents for edible oil bleaching.

Mesoporous molecular sieves, with pore sizes ranging from 2 to 50 nm, possess high surface areas, tunable pore structures, and ordered channels. Among them, MCM-41 is a representative Mobil Composition of Matter No. 41 material with a hexagonal pore structure synthesized via a surfactant-templating approach [24]. Compared to traditional microporous zeolites, the larger mesopores of the mesoporous molecular sieve MCM-41 facilitate the diffusion and reaction of medium- and large-molecular-weight organic compounds within the pore channels, thereby avoiding severe diffusion limitations. Furthermore, compared to conventional porous silica gels and activated carbon, MCM-41 possesses a more ordered pore structure, and its pore size can be finely tuned by adjusting the template agent, silicon source, and synthesis conditions [25]. Compared to other mesoporous silicas such as SBA-15 and KIT-6, MCM-41 typically exhibits stronger pore confinement effects and higher available internal and external surface areas [26].The incorporation of heteroatoms such as Al or Ti introduces Brønsted and Lewis acid sites, enhancing interaction with polar molecules and adsorption selectivity. For example, Al-MCM-41 has been applied in antibiotic adsorption and dye removal [27,28], while further amino functionalization has expanded its adsorption applications, enabling the enrichment of chlorogenic acid from plant extracts and the capture of oxyanion pollutants such as Mo(VI) from aqueous systems [29,30]. However, although Al-MCM-41 has been widely investigated in aqueous-phase adsorption and catalytic systems, its direct application in vegetable oil bleaching has rarely been addressed. More importantly, the relationship between Si/Al-regulated pore wall acidity and multi-component adsorption behavior in edible oils remains unclear.

In this study, a series of MCM-41 mesoporous molecular sieves with tunable Si/Al ratios were synthesized and employed to bleach rapeseed oil. The adsorption performance of these adsorbents toward pigments such as chlorophyll and carotenoids (lutein and β-carotene) and the retention of micronutrients including tocopherols, phytosterols and squalene during the bleaching process in rapeseed oil were investigated. Furthermore, by comparing the adsorption differences in pigments and homologous micronutrients over MCM-41 materials with different Si/Al ratios, together with kinetic, isotherm, and thermodynamic analyses of pigment adsorption, the adsorption mechanisms of pigments and micronutrients were studied. Finally, the industrial application potential of these materials was evaluated in terms of oil quality and regeneration performance. This study provides theoretical support for the development of efficient and recyclable MCM-41 adsorbents for green oil refining.

## 2. Materials and Methods

### 2.1. Materials

Crude rapeseed oil for bleaching was purchased from Yueyang, Hunan (China). To minimize matrix variation among treatments, all experiments were conducted using the same homogenized batch of crude rapeseed oil. Unless otherwise stated, all reagents were of analytical grade. Activated clay (AC), tetraethyl orthosilicate (TEOS), sodium aluminate, cetyltrimethylammonium bromide (CTAB), isooctane, N,O-bis(trimethylsilyl)trifluoroacetamide with 1% trimethylchlorosilane (BSTFA + 1% TMCS), and 2-bromoacetophenone were obtained from Shanghai Macklin Biochemical Co., Ltd. (Shanghai, China). KOH, NaOH, absolute ethanol, ammonia solution, n-hexane, methyl tert-butyl ether, anhydrous sodium bisulfate, acetonitrile, methanol, acetic acid, nitric acid, and acetone were purchased from Sinopharm Chemical Reagent Co., Ltd. (Shanghai, China). Triethanolamine was purchased from Aladdin (Shanghai, China). β-Carotene (≥95.0%), lutein (≥98.0%), cholesterol (≥99.0%), methyl heptadecanoate (≥99.0%), and a 37-component FAME standard mixture (≥99.0%) were obtained from Shanghai Yuanye Bio-Technology Co., Ltd. (Shanghai, China). Tocopherol standards (α-, β-, γ-, and δ-tocopherol, ≥98%) and methyl heptadecanoate (≥99.0%) were purchased from Shanghai APICMO Pharmaceutical Co., Ltd. (Shanghai, China).

### 2.2. Synthesis of Al-MCM-41

Following Boukoussa et al. with minor modifications [31], MCM-41 materials with different Si/Al ratios were synthesized. Reagent amounts were calculated based on the molar composition 2 SiO_2_: 0.24 CTAB: 0.5 NaOH: × Al_2_O_3_: 3 EtOH: 200 H_2_O. CTAB was mixed with distilled water and ethanol and stirred at 35 °C for 15 min. NaOH and a variable amount of NaAlO_2_ (to tune Si/Al) were then added and stirred for 10 min, maintaining pH = 11. TEOS was added dropwise, and the mixture was aged at 40 °C for 3 h. The gel was transferred to an autoclave and hydrothermally treated at 150 °C for 10 h. After cooling to room temperature, the solids were filtered and washed with deionized water to neutral pH, dried at 80 °C, and calcined at 550 °C for 6 h (heating rate 1 °C/min) to obtain Na-MCM-41 or Al-doped Na-MCM-41. The samples were ion-exchanged in 1 mol/L NH_4_NO_3_ under reflux three times, followed by washing, drying, and recalcination to yield H-MCM-41 or Al-doped H-MCM-41 (Al-MCM-41).

Seven samples were prepared: Na-MCM-41, H-MCM-41, and Al-MCM-41 (Si/Al = 80, 60, 40, 25, 15), denoted as NM, HM, AM80, AM60, AM40, AM25, and AM15, respectively.

### 2.3. Characterization

X-ray diffraction analysis (XRD): Powder diffraction analysis of the material was performed using a D8 ADVANCE X (Bruker, Karlsruhe, Germany) instrument. The test conditions were as follows: a copper target was used at a 2θ angle of 1–10°, with a scanning rate of 1°/min and an increment of 0.02°.

FT-IR spectroscopy: Fourier transform infrared (FT-IR) spectra were collected on a Nicolet IS50 FT-IR spectrometer (Thermo Fisher Scientific, Waltham, MA, USA) in transmission mode over 4000–400 cm^−1^ with a resolution of 4 cm^−1^. Prior to analysis, the solid samples were vacuum-dried at 80 °C for 12 h and stored in a desiccator.

N_2_ adsorption–desorption: N_2_ adsorption–desorption isotherms were measured using an ASAP 2460 surface area and porosimetry analyzer (Micromeritics Instrument Corp., Norcross, GA, USA). The samples were degassed at 200 °C for 8 h, and measurements were conducted at 77 K over a relative pressure range of P/P_0_ = 0–1.

NH_3_-TPD: NH_3_ temperature-programmed desorption was performed on an AutoChem HP 2950 system (Micromeritics Instrument Corp., Norcross, GA, USA). The samples were heated from room temperature to 350 °C at 10 °C/min and held for 30 min, purged with He (30 mL/min) for 1 h, cooled to 70 °C, and saturated with 10% NH_3_/He for 1 h. After switching to He (30 mL/min) and purging for 1 h to remove weakly physisorbed NH_3_, desorption was conducted by heating to 700 °C at 10 °C/min under He, with the effluent monitored by a TCD.

FE-SEM: Morphologies were observed using a field-emission scanning electron microscope (FE-SEM SU8010, Hitachi High-Tech, Tokyo, Japan). Before SEM observation, the powder samples were fixed on conductive adhesive tape and sputter-coated with gold. SEM images were obtained at an accelerating voltage of 50 kV.

### 2.4. Preparation of Bleached Oil

To minimize oxygen-induced oxidation during bleaching and to focus on adsorption-controlled pigment removal, all the bleaching experiments in this study were conducted under vacuum conditions. Briefly, 10 g of pre-bleaching rapeseed oil (PRO) was mixed with 1–5 wt% adsorbent (AC or MCM-41) and stirred at 50–100 °C for 1–35 min. After cooling, the mixture was centrifuged and filtered to obtain the bleached oil. The PRO was used as the control.

### 2.5. Pigment Analysis

#### 2.5.1. Total Bleaching Efficiency

PRO was first subjected to full-wavelength scanning using a UV–visible spectrophotometer to identify the maximum absorption wavelength [32]. After bleaching with the MCM-41 series adsorbents, the absorbance of the resulting oil samples was measured at this wavelength, and the bleaching efficiency was calculated using Equation (1). N-hexane was used as the blank.

(1) $$\mathrm{Bleaching}\;\mathrm{efficiency}\;\left(\%\right)=\frac{A_{0}-A_{1}}{A_{0}}\times 100\%$$

In the equation, A_0_ and A_1_ denote the absorbance of PRO and bleached oil, respectively.

#### 2.5.2. Lutein and β-Carotene in Bleached Oils

Calibration curves were prepared with standards at 0.05, 0.25, 0.50, 1.00, 5.00, 10.00, and 25.00 μg/mL. The linear equations were: lutein, y = 1.4414x + 0.1487 (R^2^ = 0.9965); β-carotene, y = 2.6688x − 0.0505 (R^2^ = 0.9999). Sample pretreatment followed Lu et al. [33]. Briefly, 100 mg of rapeseed oil was mixed with 6 mL methanol/ethyl acetate/ethanol (5:3:2, *v*/*v*/*v*) in a 10 mL centrifuge tube, then frozen at −20 °C for 48 h. The crystallized oil was removed by rapid vacuum filtration. The filtrate was vacuum-evaporated to dryness at 25 °C (rotary evaporator; Shanghai Yarong Biochemical Instrument Factory, Shanghai, China), reconstituted with ethyl acetate to 150 μL, and filtered through a 0.22 μm membrane. Carotenoids were separated and identified according to Zheng et al. [34] using an Ultimate 3000 UPLC-DAD system (Thermo Fisher Scientific, Waltham, MA, USA) with an Acclaim C30 column (4.6 mm × 150 mm, 3 μm; Thermo Fisher Scientific, Waltham, MA, USA).

#### 2.5.3. Total Carotenoids

A total of 0.5 g of oil (±1 mg) was dissolved in 5 mL n-hexane, and absorbance at 445 nm was measured in a 1 cm cuvette using a UV–Vis spectrophotometer (UV-VIS 1800, Shimadzu Corporation, Kyoto, Japan), with n-hexane as a blank.

#### 2.5.4. Chlorophyll Content

A 1 g portion of unbleached or bleached rapeseed oil sample was accurately weighed (to 0.0001 g) and diluted to 6 mL. The absorbance of the diluted solution was measured at 630, 670, and 710 nm using a UV–Vis spectrophotometer [20], and the chlorophyll content was calculated according to Equation (2).

(2) $$\mathrm{chlorophyll}\;\mathrm{content}\;(\mathrm{mg}/\mathrm{kg})=\frac{\left[A_{670}\;-\;\frac{A_{630}+A_{710}}{2}\right]\;V}{0.0964\;m}$$

In the equation, m is the mass of the oil sample (g), and V is the final volume of the diluted sample solution (mL); the constant 0.0964 is the conversion coefficient for calculating chlorophyll pigments.

### 2.6. Physicochemical Indices

The acid value (AV) and peroxide value (POV) were determined according to the Chinese National Food Safety Standards GB 5009.229-2025 [35] and GB 5009.227-2023 [36], respectively.

### 2.7. Content of Minor Components

#### 2.7.1. Determination of Free Fatty Acids (FFAs)

FFAs were determined according to Gao et al. [37] using an Ultimate 3000 UPLC-DAD system (Thermo Fisher Scientific, Environics, USA) with a ZORBAX SB-Aq C18 column (250 mm × 4.6 mm, 5 μm; Agilent Technologies). Detection was at 246 nm. The mobile phase was 80% methanol (A) and 20% acetonitrile: water (1:1, *v*/*v*; B) under isocratic elution with a constant mobile phase ratio (80A:20B) for 20 min. Quantification was based on the internal standard methyl heptadecanoate.

#### 2.7.2. Determination of Tocopherols

Tocopherols were analyzed following Liu et al. [38] with modifications. Standards (1.0–100.0 μg/mL) were used to construct calibration curves: α, y = 0.0992x + 0.0159 (R^2^ = 0.9997); γ, y = 0.2006x + 0.0414 (R^2^ = 0.9997); δ, y = 0.1412x − 0.0195 (R^2^ = 0.9999). The samples were 10× diluted with n-hexane. Analyses were performed on an Ultimate 3000 HPLC (Thermo Fisher Scientific, Environics, USA) with an Acclaim C30 column (250 mm × 4.6 mm, 3 μm). Mobile phases: methanol (A) and water (B); 25 °C, 0.8 mL/min, 20 μL sample volume, detection at 294 nm. Gradient: 96:4 (A/B) for 20 min, 100% A for 4 min, then back to 96:4 for 6 min.

#### 2.7.3. Determination of Phytosterols and Squalene

Based on Jiang et al. with modifications [39], the oil (0.25 g) was spiked with 200 μL 0.1% cholesterol (internal standard) and saponified with 2 mL 0.5 mol/L KOH in ethanol at 80 °C for 30 min (vortex every 10 min). After cooling, unsaponifiables were extracted with n-hexane (1 mL) and water (1.5 mL), centrifuged (4000 rpm, 10 min), and the hexane phase was collected (×3), combined, and dried under N_2_. The residue was derivatized with 150 μL BSTFA:TMCS (99:1) at 70 °C for 30 min, filtered (0.22 μm), and 1 μL injected into a GC (Agilent 6890N; Agilent Technologies, Santa Clara, CA, USA). GC analysis was performed using an Agilent Technologies 6890N gas chromatograph equipped with an HP-5MS capillary column (25 m × 0.53 mm × 0.25 μm; Agilent Technologies, Santa Clara, CA, USA). The injector temperature was set at 300 °C, and the injection volume was 1 μL with a split ratio of 15:1. High-purity nitrogen was used as the carrier gas at a constant flow rate of 1.0 mL/min. The detector temperature was maintained at 300 °C. The oven temperature program was as follows: the initial temperature was held at 200 °C for 3 min, increased to 220 °C at 2 °C/min, then increased to 300 °C at 20 °C/min and held for 15 min.

#### 2.7.4. Determination of Residual Al in Bleached Rapeseed Oil

According to the referenced method, the residual Al content in the bleached rapeseed oil was quantitatively determined using inductively coupled plasma mass spectrometry (ICP-MS, Agilent 7850, Agilent Technologies, Santa Clara, CA, USA) [40]. The ICP-MS operating conditions were as follows: RF power, 1.50 kW; sample rinse time, 30 s; auxiliary gas flow rate, 1.00 L/min; nebulizer gas flow rate, 0.7 L/min; plasma gas flow rate, 12.0 L/min; and limit of detection, 0.1 mg/kg. The Al content in the oil samples was calculated according to Equations (3) and (4).

(3) $$C_{x}=\frac{C_{0}\times f\times V_{0}\times 10^{-3}}{m\times {\;10}^{-3}}=\frac{C_{1}\times {\;V}_{0}\times 10^{-3}}{m\times {\;10}^{-3}}$$

(4) $$W=\frac{C_{x}}{10^{6}}\times 100\%$$

where *m* is the mass of the analyzed sample (g), *V_0_* is the volume of the digested sample solution (mL), *f* is the dilution factor, *C_0_* is the concentration of the element in the test solution (mg/L), *C_1_* is the concentration of the element in the original digested sample solution (mg/L), *C_x_* is the final measured result of the target element (mg/kg), and *W* is the final measured result of the target element (%).

### 2.8. Kinetic Experiments and Model Fitting

At an adsorbent dosage of 3% (g/g) and 70 °C, the rapeseed oil was bleached for 0.5, 1, 3, 5, 10, 15, 20, 25, 30 and 35 min. Chlorophyll and carotenoid contents were measured to determine the equilibrium time, and kinetics were fitted using common nonlinear models.

(5) $$q_{t}=\frac{\left(C_{0}-C_{t}\right)\times m_{0}}{m1}$$

(6) $$q_{e}=\frac{\left(C_{0}-C_{e}\right)\times {\;m}_{0}}{m1}$$

where *C_0_*, *C*_t_ and *C_e_* are concentrations in oil at initial, time t, and equilibrium (mg/kg), *m_0_* is oil mass (kg), *m_1_* is adsorbent mass (g), and *q_t_* and *q_e_* are adsorption capacities (mg/g).

(7) $$\text{Pseudo-first-order (PFO)}:\;q_{t}=q_{e}\left(1-e^{-k_{1}t}\right)$$

(8) $$\text{Pseudo-second-order (PSO)}:\;q_{t}=\frac{q_{e}^{2}k_{2}t}{1+q_{e}k_{2}t}$$

where *k_1_* (min^−1^) and *k_2_* (g∙mg^−1^∙min^−1^) are rate constants, and *t* is time (min).

### 2.9. Adsorption Isotherm Fitting

At 70 °C for 20 min, the rapeseed oil was bleached using MCM-41 series materials at 1, 2, 3, 4 and 5% (g/g). Isotherms for chlorophyll and carotenoids were fitted with:

(9) $$\mathrm{Langmuir}:\;q_{e}=\frac{k_{L}q_{m}C_{e}}{\left(1+k_{L}C_{e}\right)}$$

(10) $$\mathrm{Freundlich}:\;q_{e}=k_{F}C_{e}^{\frac{1}{n}}$$

where *q_m_* is the monolayer capacity (mg/g), *k_L_* is the Langmuir constant, and *k_F_* and *1/n* describe adsorption strength and surface heterogeneity.

### 2.10. Thermodynamic Experiments and Fitting

With 3% (g/g) adsorbent and 20 min contact time, bleaching was conducted at 50, 60, 70, 80, 90 and 100 °C. Thermodynamic parameters were calculated by:

(11) $$k_{d}=q_{e}/C_{e}$$

(12) $$∆G^{0}=∆H^{0}-T∆S^{0}$$

(13) $$∆G^{0}=-RT\ln\left(k_{d}\right)$$

where ∆G^0^ is the Gibbs free energy change (kJ/mol), ∆H^0^ is the enthalpy change (kJ/mol), ∆S^0^ is the entropy change (kJ∙mol^−^1∙K^−1^), R is the universal gas constant (8.314 × 10^−3^ kJ∙mol^−^1∙K^−1^), T is the absolute temperature (K), and k_d_ is the adsorption distribution coefficient.

### 2.11. Adsorbent Recycling Experiments

(1) Elution and regeneration

After the initial bleaching of the rapeseed oil, the spent adsorbent was regenerated by solvent washing and ultrasonication to remove the adsorbed pigments, impurities, and residual oil. After repeated washing, the adsorbent was dried in a vacuum oven and then reused for adsorption tests following the same procedure described above.

(2) Determination of oil recovery

The bleached oil and the residual oil were collected and calculated according to Equation (14):

(14) $$\mathrm{oil}\;\mathrm{recovery}\;(\%)\;=\;\frac{m_{1}\;+{\;m}_{2}}{m}\;\times \;100$$

where m_1_ is the mass of the bleached oil (g), m_2_ is the mass of the residual oil eluted from the adsorbent (g), and m is the mass of the rapeseed oil prior to bleaching (g).

### 2.12. Statistical Analysis

All the experiments were conducted in triplicate, and the results are expressed as mean ± standard deviation. Statistical analysis was performed using SPSS Statistics 27 (IBM Crop., Armonk, NY, USA), and the experimental results were evaluated using Duncan’s multiple range test. Data visualization was carried out using Origin 2021 (OriginLab, Northampton, MA, USA).

## 3. Results and Discussion

### 3.1. Characterization of the Synthesized Adsorbents

**Figure 1:**
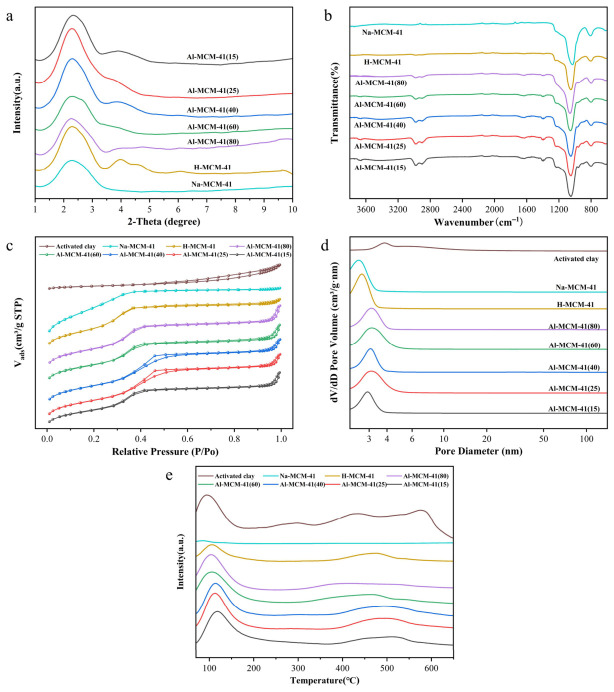
Characterization of MCM-41 series and activated clay. XRD (**a**), FT-IR (**b**), N_2_ adsorption–desorption curve (**c**), pore-size distribution (**d**) and NH_3_-TPD (**e**).

Small-angle XRD is a key technique for evaluating the mesostructural ordering of MCM-41-type materials. The presence, position, and resolution of the characteristic reflections provide information on hexagonal pore ordering, long-range structural regularity, and changes in unit-cell spacing. As shown in Figure 1a, all MCM-41 samples displayed an intense primary peak at 2.1–2.4° indexed to the (100) plane, together with weaker (110) and (200) reflections at 3.5–5.0°, indicating that Na-MCM-41, H-MCM-41, and Al-MCM-41 with different Si/Al ratios all retained the typical two-dimensional hexagonally ordered mesoporous framework of MCM-41. After Al incorporation, the (100) peak became relatively broader and the (110)/(200) reflections less resolved, suggesting that Al introduction disturbed the long-range ordering of the siliceous framework and induced slight pore channel distortion [30]. Nevertheless, the distinct (100) peak indicates that the mesoporous structure remained preserved. Moreover, with decreasing Si/Al ratio, particularly at Si/Al = 15, the d(100) peak shifted slightly to higher angles, implying reduced interplanar spacing and unit-cell parameter, likely due to pore wall densification and a slight contraction of pore size and interpore distance.

FT-IR spectroscopy was used to characterize the framework integrity, surface functional groups, and the formation of acidic sites in the MCM-41 samples. All the samples exhibited characteristic Si–O–Si stretching vibrations at approximately 1080 and 800 cm^−1^ (Figure 1b), indicating that Na-MCM-41, H-MCM-41, and Al-MCM-41 with different Si/Al ratios retained the mesoporous siliceous framework. Weak bands in the 2800–3000 cm^−1^ region were assigned to the stretching vibrations of aliphatic C–H groups, mainly from –CH_2_– and –CH_3_ groups. These bands may originate from trace residual CTAB-derived organic species or adsorbed hydrocarbons after calcination. A broad band at 3600–3000 cm^−1^ and a bending vibration peak at 1630 cm^−1^ were observed, which are attributed to surface silanol groups and adsorbed water molecules within the pores. Compared with Na-MCM-41, H-MCM-41 and Al-MCM-41 showed stronger absorption around 3600 cm^−1^, suggesting an increased density of surface hydroxyl groups. With decreasing Si/Al ratio from 80 to 15, the broad band at 3600–3000 cm^−1^, the sharp peak at approximately 3690 cm^−1^, and the defect band at 960 cm^−1^ all became more intense, indicating the successful incorporation of Al into the siliceous framework, leading to the formation of more Si–OH and Si–OH–Al species and a higher density of acidic hydroxyl and defect sites [41]. These sites can provide hydrogen-bonding and acid–base interaction centers for polar pigment molecules, particularly pheophytin/chlorophyll derivatives containing carbonyl and ester groups. As a result, both the acidity and hydrophilicity of the materials were enhanced, providing a structural basis for their bleaching performance.

According to the updated IUPAC classification of physisorption isotherms, the N_2_ adsorption–desorption isotherms of the MCM-41 series can be described as Type IV(a) isotherms with H1-type hysteresis loops, showing a distinct capillary condensation step at P/P_0_ = 0.2–0.6. These features are characteristic of mesoporous materials with relatively uniform pore channels (Figure 1c). These features confirm that the materials possessed uniform mesoporous structures with a relatively concentrated pore-size distribution of approximately 2–4 nm (Figure 1d). This pore scale is well matched with the molecular dimensions of the major pigments, including chlorophyll (~2.5 nm), lutein (~3.00 nm), and β-carotene (~2.80 nm), which facilitates the diffusion of pigment molecules into the pores and promotes confinement-enhanced contact in the oil phase [42,43,44,45]. In contrast, activated clay showed a gradual adsorption increase over the whole pressure range and a broad, tailing hysteresis loop at P/P_0_ > 0.4, with pore sizes extending from approximately 3 nm to tens of nanometers, indicating a disordered porous structure with a much broader pore-size distribution. Na-MCM-41 exhibited the highest specific surface area (1048 m^2^/g) and a pore volume of 0.74 cm^3^/g (Table 1), indicating that the unmodified material retained the most intact framework and the highest degree of mesostructural order. After ammonium exchange and calcination, the specific surface area of H-MCM-41 decreased to 881.9 m^2^/g, whereas the pore volume slightly increased to 0.77 cm^3^/g, which may be related to changes in the pore wall chemical environment [46]. After Al incorporation, the specific surface area and pore volume of Al-MCM-41(80-25) gradually decreased, indicating that moderate Al incorporation increased framework density without destroying the ordered mesostructure. By contrast, Al-MCM-41(15) showed a more pronounced decrease in both specific surface area (698 m^2^/g) and pore volume (0.70 cm^3^/g), suggesting that excessive Al loading led to reduced mesostructural order, possibly accompanied by pore narrowing, extra-framework Al species, or local framework collapse [47].

NH_3_-TPD profiles (Figure 1e) were further used to evaluate the acidity of the materials. In general, the desorption peak area reflects the amount of acid sites, whereas the desorption temperature is associated with acid strength [48]. Activated clay exhibited weak and strong acid desorption peaks at 94.28 °C and 576.26 °C, respectively, together with a high total acidity of 0.8564 mmol/g. In contrast, Na-MCM-41 showed negligible acidity, with a total acid amount of only 0.024 mmol/g [49]. The sodium mainly originates from the sodium-containing precursors used in the synthesis system and is predominantly present as extra-framework, exchangeable cations distributed near the inner surface of the mesopore walls. In the non-ammonium-exchanged Al-MCM-41, these Na^+^ species primarily serve to compensate for the negative charge introduced by tetrahedrally coordinated framework Al and can be further replaced by H^+^ during subsequent ion-exchange treatment, thereby generating Brønsted acid sites such as Si-OH-Al [50]. After NH_4_^+^ exchange and calcination, the acidity of H-MCM-41 increased to 0.2024 mmol/g. With further Al incorporation, the total acidity of Al-MCM-41 80–25 increased to 0.3557–0.5502 mmol/g, while the high-temperature desorption peak shifted to 416.42–496.62 °C, indicating simultaneous increases in both acid site density and acid strength. For Al-MCM-41(15), the total acidity was 0.5561 mmol/g, which was very close to that of Al-MCM-41(25) at 0.5502 mmol/g. This indicates that further decreasing the Si/Al ratio from 25 to 15 did not substantially increase the total number of acid sites. However, the second NH_3_ desorption peak shifted from 496.62 °C for AM25 to 509.89 °C for AM15, suggesting a slight increase in acid strength.

### 3.2. Bleaching Performance

#### 3.2.1. Total Bleaching Efficiency

**Figure 2:**
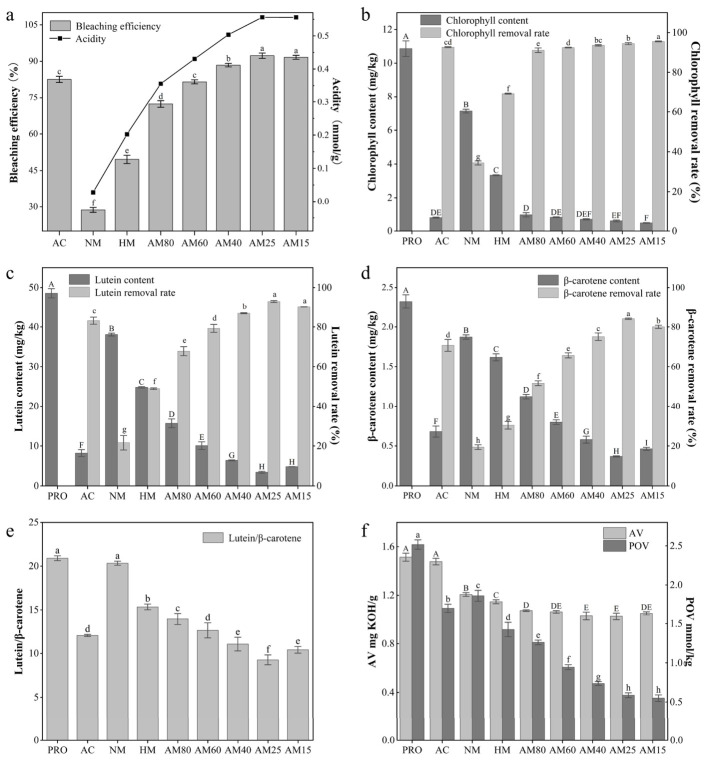
The bleaching performance of rapeseed oil using the MCM-41 series and AC adsorbents. The total bleaching efficiency of rapeseed oil (**a**); the removal efficiency of chlorophyll (**b**); the removal efficiency of lutein (**c**); the removal efficiency of β-carotene (**d**); the changes in lutein/β-carotene ratio (**e**); the changes in AV and POV (**f**). Note: Different uppercase and lowercase letters indicate significant differences following Duncan’s test, with *p* < 0.05. PRO denotes pre-bleaching rapeseed oil; AC denotes activated clay; NM denotes Na-MCM-41; HM denotes H-MCM-41; and AM80, AM60, AM40, AM25 and AM15 denote Al-MCM-41 with Si/Al ratios of 80, 60, 40, 25 and 15, respectively. Total bleaching efficiency (%) was calculated as the percentage reduction in absorbance of the bleached oil compared with the crude rapeseed oil.

Under the same conditions (70 °C, 20 min, and 3 wt% adsorbent dosage), the bleaching efficiency of the MCM-41 series increased with increasing acidity except for AM15 (Figure 2a), because AM15 showed a total acidity comparable to AM25 but a slightly higher acid strength, while its mesopore regularity, pore size, and specific surface area decreased, which limited pigment diffusion and adsorption. Among the MCM-41 series, NM exhibited the lowest bleaching efficiency at only 28.70%, while the bleaching efficiency of HM was 49.58%, indicating a significant enhancement in bleaching efficiency after the acidification of MCM-41. Further, the bleaching efficiencies of AM80–AM25 after Al component was gradually added to HM were from 72.26% to 92.30% with the acid amount increasing from 0.3557 to 0.5502 mmol/g, the pore diameter decreasing slightly from 4.0 to 3.8 nm and specific surface area from 813 to 777 m^2^/g, while the bleaching efficiency of AM15 decreased slightly to 91.65% with the acid amount reaching saturation (0.5561 mmol/g) and the pore diameter (3.7 nm) and specific surface area (698 m^2^/g) decreasing markedly. These indicate that bleaching performance showed a dependence on acidity, but was also jointly governed by pore structure, site accessibility, and surface heterogeneity, which is consistent with previous reports showing that pigment adsorption on montmorillonite-based clays is simultaneously affected by the number and strength of acidic sites as well as pore-size distribution [51]. As a control, the bleaching efficiency of AC was 82.58%, indicating that low-Si/Al-ratio Al-MCM-41 adsorbents (Si/Al = 40–15) with ordered mesoporous structures and high specific surface areas exhibited stronger bleaching efficiency than AC for rapeseed oil.

#### 3.2.2. Removal Efficiency of Pigment Components

Previous studies have reported that chlorophyll in rapeseed oil mainly exists as pheophytin a [23], and the chlorophyll content determined in this study by spectrophotometry was 10.86 mg/kg. In addition, UPLC analysis showed that the major carotenoids in crude rapeseed oil before bleaching were lutein (48.56 mg/kg) and β-carotene (2.52 mg/kg). Under bleaching conditions (70 °C, 20 min, 3% dosage, vacuum), for chlorophyll (Figure 2b), Al-free MCM-41 exhibited much lower removal, although HM (69.28%) was markedly more effective than NM (34.38%). In the Al-doped series, AM80 achieved 91.12% chlorophyll removal, comparable to AC, while AM25–AM15 reached 94.46–95.57%. AC already showed lower removal efficiency (92.67%). Combined with acidity characterization, these results indicate that H and Al introduction simultaneously increased both acid amount and acid strength in MCM-41, thereby markedly enhancing chlorophyll adsorption. In contrast, lutein (Figure 2c) and β-carotene (Figure 2d) removal increased progressively with decreasing Si/Al ratio and reached a maximum at AM25. The removal efficiencies of AC for lutein and β-carotene were 83.13% and 70.73%, respectively, both significantly lower than those of AM25 (92.94% and 84.09%); this can be attributed to the narrow pore-size range of AM25 (2–4 nm), which better matches the molecular dimensions of lutein and β-carotene, thus generating stronger confinement, while its high surface area provides greater van der Waals contact area [52]. In addition, lutein and β-carotene are long π-conjugated molecules with high polarizability, and the strong acid sites introduced by Al doping can induce instantaneous dipoles, thereby strengthening dispersion interactions and promoting multipoint contact with the pore walls [53].

Both AC and the MCM-41 series showed the same removal order: chlorophyll > lutein > β-carotene. As a free-base chlorin formed after demetallation, pheophytin a can be protonated at acidic sites to form cationic species [54]. The positive charge is delocalized over the conjugated chlorin macrocycle, which strengthens its interaction with acidic surfaces. Meanwhile, the 13^1^-keto and 13^2^-methoxycarbonyl groups on the E ring can form hydrogen bonds with surface Si–OH or Si–OH–Al groups, further enhancing adsorption [55]. Although lutein and β-carotene are structurally related carotenoids, the terminal hydroxyl groups of lutein can form hydrogen bonds with surface hydroxyls, whereas β-carotene shows a weaker response to acid sites. Therefore, β-carotene was removed less efficiently than lutein, indicating that pigment removal was closely related to molecular structure.

In addition, the lutein/β-carotene ratio in bleached oils obtained with the MCM-41 series gradually decreased with decreasing Si/Al ratio, but increased significantly at AM15 (Figure 2e). NM showed almost no surface acidity and thus did not differ significantly from PRO, whereas from AM80 to AM25, decreasing Si/Al rapidly increased acidity and strengthened the adsorption potential of the pore walls [41], thereby enhancing pigment removal. In AM15, however, the amount of medium-strong acid sites decreased relative to AM25, while excessive Al incorporation caused pore shrinkage and mass-transfer limitation [56]. Under these conditions, β-carotene, which depends more strongly on dispersion interaction and pore confinement, rebounded more markedly, resulting in an increased ratio.

### 3.3. Effect of MCM-41 on Physicochemical Properties of Oil

Acid value (AV) and peroxide value (POV) are important indicators for evaluating vegetable oil quality, reflecting the degrees of hydrolytic and oxidative deterioration, respectively, and are closely related to storage stability and edible quality.

Under bleaching conditions of 70 °C, 20 min, 3% dosage, and vacuum, the effects of the MCM-41 series and activated clay (AC) on the physicochemical properties of rapeseed oil were evaluated (Figure 2f). The MCM-41 adsorbents reduced AV significantly more than AC, whereas AC contributed little to AV removal. For MCM-41, the acid value gradually decreased with the introduction of H and Al, except for AM15. Among MCM-41 adsorbents, HM decreased AV from 1.51 to 1.21 mg KOH/g, and after Al incorporation, AV further declined from 1.07 mg KOH/g to 1.02 mg KOH/g with decreasing the Si/Ai ratio from 80:1 (AM80) to 25:1 (AM25), and then increased by 1.05 mg KOH/g at the 15:1 Si/Ai ratio (AM15). When the NH_3_-TPD of MCM-41 was analyzed (Table 1), the results suggested that the MCM-41 series possessed milder acid strength, with a second peak temperature from 416 to 510 °C for AM80-15, and a much lower acidity amount calculated from the peak area [48] from 0.3557 to 0.5561 mmol/g for AM80-15, compared to AC with a 576 °C second peak temperature and 0.8564 mmol/g acidity amount, indicating that the AM series with the lower acid strength from 0.3557 to 0.5561 mmol/g was more favorable than AC for the synchronous removal of FFAs with pigments in oil, because moderate acid strength favors hydrogen bond formation between adsorbents and FFAs, thereby promoting adsorption, whereas excessive strong acid sites may accelerate oil hydrolysis and generate new FFAs [15,18].

For POV (Figure 2f), NM decreased the POV of PRO from 2.52 to 1.86 mmol/kg after bleaching, and HM from the acidification of NM was further reduced to 1.42 mmol/kg. With the treatment of AM from Al incorporation of HM, the POV decreased to 1.26–0.55 mmol/kg for AM80–15 as the Si/Al ratio gradually decreased., indicating that the low-Si/Al-ratio MCM-41 (AM25–AM15) were more favorable for peroxide removal, because the lower Si/Al ratio increased the Si-OH of MCM-41 (Figure 1b) and thereby strengthened dipole interactions and hydrogen bonding between the peroxides and the pore walls of the AM materials. By contrast, AC only reduced the POV to 1.70 mmol/kg. This may be because of the presence of trace transition metals, such as Fe and Cu, that can catalyze peroxide formation and thus offset its apparent removal efficiency [57].

### 3.4. Minor Components

Minor components in vegetable oils, such as Tocopherols, phytosterols, squalene and FFAs, are closely associated with oxidative stability and also exert important nutritional and physiological functions. Thus the retention of tocopherols, phytosterols, and squalene was used to evaluate the adsorption selectivity of the adsorbents during bleaching. In this context, an adsorbent with high selectivity should remove pigments efficiently while retaining these lipid-soluble micronutrients at high levels.

#### 3.4.1. Tocopherols

The total tocopherol content in PRO was 652.14 mg/kg, with α-, γ- and δ-tocopherol contents of 255.92, 381.04 and 15.17 mg/kg respectively (Table 2). The MCM-41 series reduced total tocopherols in oils after bleaching PRO at 70 °C for 20 min; the retention rates of total tocopherols ranged from 93.72 to 95.46%, indicating that MCM-41 exhibits a high retention of tocopherol under vacuum bleaching conditions. In contrast, AC significantly reduced total tocopherols to 567.47 mg/kg, with a retention of 87.02%. The greater loss with AC is mainly attributed to its high density of acidic sites and trace Fe and Cu, which promote the strong adsorption of tocopherols and catalyze their oxidation to quinone products [58].

A comparison of the retention of each tocopherol across different adsorbents further showed that α-tocopherol was retained most effectively in NM (91.48%), decreased to 90.08% in HM, declined further from 88.49% to 87.45% in AM80–AM40, and then slightly recovered to 87.87% and 88.67% in AM25 and AM15, respectively. The slight rebound of α-tocopherol retention in AM25 and AM15 likely reflected the onset of pore size/surface area limitations, which partially counteracted the enhanced polarity/acidity-induced consumption observed from AM80 to AM40. By contrast, γ-tocopherol remained relatively stable across the MCM-41-based adsorbents, ranging from 97.98% to 99.27%, while δ-tocopherol also varied from 90.11% to 91.69%. These results indicate that, in HM and all AM adsorbents, the retention rates consistently followed the order γ-tocopherol > δ-tocopherol > α-tocopherol, whereas in NM, α- and δ-tocopherol showed similar retention rates. Here, NM exhibits the smallest difference in the adsorption of tocopherol analogs because it contains the fewest Si–OH groups (Figure 1b), thus it adsorbs the least amount of peroxides and causes less oxidative loss of α-tocopherol due to lower local concentrations of peroxides within the pores [59]. Meanwhile, HM from the acidification of NM and AM80–AM15 from Al incorporation with HM increased gradually the abundance of Si–OH/Si–OH–Al sites according to the intensity changes in absorbance in 960 cm-1 (Figure 1b) and acidity (Table 1) so as to enhance the adsorption difference in the three tocopherols. In addition, the lower retention of δ-tocopherol than γ-tocopherol may be related to the fewer methyl substituents and lower steric hindrance of δ-tocopherol [60], which make it more likely to interact with surface Si–OH/Si–OH–Al groups.

#### 3.4.2. Phytosterols and Squalene

The total phytosterol content in PRO was 845.47 mg/100 g, of which the contents of sitosterol, campesterol and brassicasterol were 450.02 mg/100 g, 297.41 mg/100 g, 98.04 mg/100 g, respectively (Table 2), while squalene content was 3.53 mg/100 g. Following the bleaching of MCM-41 such as NM, HM or AM80–AM15, the total phytosterol retention rate remained in the range from 97.72% to 98.83%. In contrast, AC reduced the retention rate of the three phytosterols to 96.11%, less than that in MCM-41, because AC may catalyze the dehydration condensation of phytosterols [61], thereby reducing the phytosterol retention.

From the retention of the three phytosterols by the different MCM-41 adsorbents, it was found that NM gave the lowest retention for all three phytosterols, with retention rates of 95.69% for brassicasterol, 98.37% for campesterol, and 97.73% for sitosterol. After the treatment of HM or AM80-AM15, the retention of all three phytosterols increased slightly. However, as the Si/Al ratio decreased from 80:1 (AM80) to 15:1 (AM15), phytosterol retention gradually declined, with brassicasterol decreasing from 96.83% to 96.22%, campesterol from 99.35% to 98.86%, and sitosterol from 98.93% to 97.67%. In addition, the retention of the three phytosterols consistently followed the order of campesterol > sitosterol > brassicasterol for all MCM-41 adsorbents, maybe because NM, with the largest specific surface area and the smallest pore diameter, imposed the strongest physical retention on sterol molecules. After acidification to form HM and further Al incorporation to obtain AM, the pore diameter increased while the specific surface area decreased, and the number of surface Si–OH/Si–OH–Al sites increased. As a result, the increased hydrophilicity of HM and AM80-AM15 relative to NM reduces the entrapment of hydrophobic phytosterols within the pores, thereby improving the phytosterol retention rate. However, phytosterol retention lowered with decreasing the Si/Al ratio of AM80-AM15, maybe because the increase in strong acid sites caused by the decrease in the Si/Al ratio induced phytosterol dehydration [62]. Furthermore, the retention of brassicasterol was consistently lower than that of campesterol and sitosterol, likely because brassicasterol contains both a Δ^5^ double bond in the sterol nucleus and a Δ^22^ double bond in the side chain, which may enhance electron delocalization, stabilize carbocation intermediates, and lower the activation barrier for acid-catalyzed dehydration of the 3β-OH group. Moreover, as the C24-ethyl-substituted sitosterol is longer than the campesterol molecule, it may be easier to be physically retained within the pores of the MCM-41 series of adsorbents, resulting in a lower retention rate than that of campesterol after adsorption.

Table 2 also shows that the retention rates of squalene in bleached rapeseed oil for the various MCM-41 adsorbents ranged from 96.03% to 99.15%, with NM exhibiting the highest retention rate, and the retention rate decreased gradually as the Si/Al ratio decreased after bleaching for AM80-15. AC as a control exhibited a squalene retention rate of 91.22%, which was lower than that of the MCM-41 series of adsorbents. This is because a decrease in the Si/Al ratio increases acid strength, and AC possesses the highest number of strong acid sites (Table 1). Under the influence of strong acid sites, the double bonds in squalene are prone to acid-catalyzed reactions such as isomerization, cyclization and oligomerization [63], thereby leading to a reduction in squalene content.

#### 3.4.3. Free Fatty Acids

Free fatty acids (FFAs) are key quality indicators of vegetable oils. UPLC analysis detected four free fatty acids such as stearic acid (C18:0), oleic acid (C18:1), linoleic acid (C18:2), and α-linolenic acid (C18:3) and no other free fatty acids were detected. After the MCM-41 series adsorption at 70 °C for 20 min with a 3% addition, the removal efficiencies of C18:0, C18:1, C18:2, and C18:3 in oil ranged from 6.45% to 17.20%, 7.45% to 30.07%, 7.12% to 28.84%, and 12.39% to 30.21%, respectively (Table 2). In addition to the hydrogen bonding between the carboxyl group and acidic sites, the C=C bonds in unsaturated FFAs increase polarizability and strengthen dispersion interactions with the adsorbent [64,65]. Therefore, the most unsaturated FFA, C18:3, exhibited the highest removal.

### 3.5. Adsorption Kinetics, Isotherms, and Thermodynamics of Chlorophylls and Carotenoids

#### 3.5.1. Adsorption Kinetics

Adsorption kinetics describe the migration of pigments from the oil phase to the adsorbent surface and pore channels until equilibrium is reached, and thus provide a basis for optimizing bleaching time, identifying rate-limiting steps, and evaluating adsorbent performance [66]. In the pseudo-first-order and pseudo-second-order models (Figure S1), *q_e_* represents the equilibrium adsorption capacity, *k_1_* the apparent rate constant, *k_2_* the rate constant associated with site occupation, and *h_0_* the initial adsorption rate. Chlorophylls and carotenoids were better fitted by the pseudo-second-order model (Table 3), as indicated by higher R^2^ values. In addition, the *k_2_* and *h_0_* values of AM40–15 were consistently higher than those of AC, suggesting that uniform mesoporous channels reduce diffusion resistance and strengthen pigment–surface interactions through confinement effects [67].

For chlorophyll, the AM series exhibited superior kinetic performance. With decreasing Si/Al ratio, both *k_2_* and *h_0_* increased continuously, reaching 42.128 and 4.947 in AM15, respectively, while *q_e_* also showed an increasing trend. This indicates that increasing acidity enhanced both the adsorption capacity and site-occupation rate of chlorophyll. Notably, this kinetic enhancement occurred despite the gradual decrease in specific surface area and pore size within the AM series. Therefore, within the suitable mesopore range of 2–4 nm, chlorophyll adsorption was more closely associated with surface acidity than with specific surface area or pore size. The increased density of acidic and polar sites promotes the interfacial enrichment of chlorophyll and accelerates site occupation [68]. In contrast, NM and HM showed only slight increases in *k_2_* and *h_0_*, indicating that protonation alone has limited influence on adsorption kinetics. Overall, the surface acidity of AM dominates chlorophyll adsorption rate.

For carotenoids, AM80 exhibited the highest *k_2_* and *h_0_* values, indicating the fastest initial adsorption rate. As the Si/Al ratio further decreased, the *k_2_* values of AM60–AM15 gradually declined, whereas *q_e_* increased from 1.169 to 1.582 mg/g. This suggests that, despite its lower acidity, AM80 retained a kinetic advantage because its larger pore size and higher surface area reduced intraparticle diffusion resistance. With further increases in pore wall acidity, more polar coexisting components in oil may preferentially occupy active sites through hydrogen bonding and alter pore transport, leading to earlier competitive adsorption and intraparticle diffusion limitations [69], and thus a slight decrease in k_2_. This competitive adsorption is reasonable because rapeseed oil contains multiple polar species, such as FFAs, peroxides, and tocopherols, which can interact with Si–OH/Si–OH–Al sites through hydrogen bonding, dipole interactions, or acid–base interactions, thereby reducing the immediate accessibility of carotenoids to the pore wall surface. Therefore, AM pore structure mainly governs carotenoid adsorption rate, whereas surface acidity mainly affects competitive behavior, mass transfer, and adsorption capacity.

#### 3.5.2. Adsorption Isotherms

Adsorption isotherms were used to characterize the equilibrium distribution of pigments between the oil phase and adsorbents at constant temperature and contact time, thereby evaluating adsorption capacity, interfacial affinity, and surface-site heterogeneity. The isotherm parameters (Figures S2 and S3) showed that the *R_L_* values of chlorophyll and carotenoids on AC and the MCM-41 series were all within 0–1 and *n* > 1 (Table 3), indicating favorable adsorption under the present conditions. In terms of model fitting, carotenoid adsorption on all MCM-41 adsorbents was better described by the Freundlich model, while chlorophyll adsorption also generally followed the Freundlich model except for NM. These results suggest that the equilibrium adsorption of the two kinds of pigments was dominated mainly by heterogeneous multisite adsorption rather than ideal monolayer coverage on a homogeneous surface [70].

For chlorophyll, the *q_m_* and *K_F_* values of the AM series increased from 0.997 mg/g and 0.328 for AM80 to 2.241 mg/g and 0.644 for AM15, respectively, as the number of total acidic sites increased with the Si/Al ratio decrease, indicating that Al incorporation and the associated increase in surface acidity for the AM series favored the enhancement of chlorophyll adsorption capacity at equilibrium. Combined with the kinetic results, these data suggest that chlorophyll adsorption was more sensitive to the number of acidic sites of the MCM-41 adsorbents. For carotenoids, AM25 exhibited the highest *q_m_*, *K_L_*, and *K_F_* values, reaching 2.853 mg/g, 0.344, and 0.983, respectively, whereas these parameters decreased slightly for AM15, indicating that carotenoid adsorption capacity in the AM series does not continuously increase with increasing AM acidity, but reaches the optimum equilibrium performance under AM25 acidity. The efficient removal of carotenoids therefore depended more strongly on the synergy among acidity, ordered mesoporous structure, and site accessibility. Overall, chlorophyll adsorption was promoted mainly by the enhancement of acidic sites, whereas carotenoid adsorption depended more on the cooperative optimization of pore structure and surface acidity.

#### 3.5.3. Adsorption Thermodynamics

Adsorption thermodynamics provides an insight into the spontaneity, temperature dependence, and interaction strength of the bleaching process in terms of energy and driving force. Overall, the ΔG^0^ values of both chlorophyll and carotenoids on MCM-41 and AC adsorbents were negative over 50–100 °C and became more negative with increasing temperature from 50 to 100 °C (Table 3 and Figure S4), indicating that the adsorption of chlorophyll and carotenoid was spontaneous and thermodynamically more favorable with temperature increases. The corresponding positive ΔH and ΔS^0^ values of chlorophyll and carotenoid further suggested that the process was endothermic and entropy-driven. The ΔH^0^ values ranged from 14.83 to 35.83 kJ/mol for chlorophyll and from 16.14 to 57.34 kJ/mol for carotenoids on MCM-41 and AC adsorbents, indicating that the adsorption of chlorophyll and carotenoid is dominated by physical adsorption [71]. Moreover, the ΔG^0^ values of the AM series were generally lower than those of NM and HM, and the adsorption driving force increased progressively with the decreasing Si/Al ratio, indicating that Al incorporation enhanced the thermodynamic affinity of the adsorbents toward chlorophyll and carotenoid through increasing the surface acidity of the MCM-41 series.

Notably, the adsorption of chlorophyll and carotenoid exhibited distinct thermodynamic responses. Carotenoids showed overall higher ΔH^0^ and ΔS^0^ values than chlorophyll in the MCM-41 series, suggesting a greater contribution from desolvation, conformational adjustment, and aggregate rearrangement, and thereby a stronger sensitivity to temperature variation [72]. Among all adsorbents, AC showed the strongest temperature dependence for carotenoid adsorption with the highest ΔH^0^ (35.83 kJ/mol) and ΔS^0^ (57.34 kJ/mol), suggesting a greater thermodynamic cost for adsorption. In contrast, chlorophyll displayed a stronger thermodynamic response on the AM series, indicating that its adsorption was more effectively promoted by enhanced pore wall polarity and surface acidity. For carotenoids, however, adsorption spontaneity depended more strongly on the balance between acid strength and intrapore accessibility, with AM25 showing the optimal thermodynamic performance because it provided both proper acidity and favorable mesopore accessibility.

#### 3.5.4. Proposed Adsorption Mechanism

Based on the FT-IR, NH_3_-TPD, pore structure characterization, and adsorption fitting results, the adsorption mechanism of pigments on Al-MCM-41 can be interpreted as a synergistic process involving surface acidity, pore confinement, and molecular-structure-dependent interactions. FT-IR analysis showed that Al incorporation enhanced the intensity of hydroxyl-related bands and the defect band associated with Si–OH/Si–OH–Al species, indicating an increased density of polar and acidic sites on the pore walls. NH_3_-TPD further confirmed that decreasing the Si/Al ratio increased the acid site amount and acid strength. These sites served as adsorption centers for pigments by providing hydrogen bonding, dipole interaction, and acid-assisted surface interaction.

Chlorophyll adsorption was mainly governed by the acidic sites. The polar carbonyl and ester groups in chlorophyll derivatives can interact with Si–OH/Si–OH–Al groups, while the conjugated macrocycle may be stabilized on acidic pore walls through protonation-related and dipole-assisted interactions. This explains why chlorophyll removal and adsorption capacity increased with increasing acidity, even when the specific surface area decreased slightly. In contrast, carotenoid adsorption depended more strongly on the balance between pore accessibility and surface acidity. Lutein can interact with surface hydroxyl groups through its terminal hydroxyl groups, whereas β-carotene mainly relies on dispersion interaction and mesopore confinement because of its nonpolar structure. Therefore, AM25 showed the optimal carotenoid removal because it provided appropriate acidity together with accessible 2–4 nm mesopores, whereas excessive Al loading in AM15 reduced pore accessibility and limited carotenoid diffusion.

The kinetic, isotherm, and thermodynamic results further support this mechanism. The pseudo-second-order model suggests that pigment uptake was closely related to the occupation of available surface sites, whereas the better fit of the Freundlich model indicates heterogeneous multisite adsorption rather than ideal monolayer adsorption. The positive ΔH^0^ values and negative ΔG^0^ values indicate that the adsorption process was spontaneous and endothermic, while the moderate ΔH values suggest that physical adsorption dominated, involving hydrogen bonding, dispersion forces, dipole interactions, and pore confinement rather than irreversible chemical bonding.

### 3.6. Regeneration and Reusability Tests

AM25 in the MCM-41 series was selected for the evaluation of its regenerative properties due to AM25 exhibiting the best bleaching performance including bleaching efficiency, micronutrient retention rate, and POV and AV changes.

#### 3.6.1. Residual Metal (Al)

Aluminum (Al) overconsumption can pose a risk to human health. The Al content in PRO from the same batch was 0.688 mg/kg, likely originating from raw materials or processing [73], well below the Provisional Tolerable Weekly Intake of 2 mg/kg bw/week set by JECFA for Al in food. After AM25 bleaching, Al decreased to below the detection limit (0.1 mg/kg) after the first bleaching cycle, indicating that AM25 can promote the absorption of Al during the bleaching process through the nanoconfinement effect and hydrogen bonding between acidic sites on the pore walls and Al complexes [74], and demonstrating the good structural stability of AM25.

#### 3.6.2. Elution Solvent Selection

For AM25, the pigment recovery rate during the first elution of AM25 is positively correlated with the second bleaching efficiency (Table 4). n-Hexane gave the highest β-carotene recovery (99.28%) but lower chlorophyll/lutein recovery (85.07% and 89.78%), resulting in the lowest second-cycle efficiency (81.04%). Ethanol improved chlorophyll/lutein recovery (98.56% and 98.17%) but reduced β-carotene recovery (86.08%), giving an intermediate efficiency (87.42%). The mixed solvent ethanol: n-hexane = 5:1 achieved a high recovery for all pigments (98.8–99.4%) and the highest re-bleaching efficiency (92.82%). These are because β-carotene is highly nonpolar and desorbs mainly via solubilization, favoring nonpolar solvents, whereas lutein is poorly soluble in hexane but more soluble in polar media [75]. Thus, hexane cannot effectively disrupt dipole/H-bond interactions between pheophytin/lutein and Si–OH/Si–OH–Al, leaving residues that limit reuse. Ethanol, as a polar protic solvent, enhances pigment partitioning and promotes desorption by competing for H-bond/dipolar interactions on the pore wall [76], but dissolves less β-carotene and residual oil. The 5:1 ethanol/hexane mixture is synergistic: ethanol releases chlorophyll/lutein from the surface sites and chamber of AM25, while hexane removes hydrophobic β-carotene and residual oil, releasing pore space and restoring accessible sites.

#### 3.6.3. Oil Recovery

After elution with the mixed solvent, oil recovery was 99.98% for AM25 and 99.84% for AC, corresponding to residual oil rates of 0.02% and 0.16% respectively, indicating more residual oil retained by AC. This is attributed to the broad pore-size distribution with micropores and irregular pores of AC, in which it is difficult to elute the oil, whereas AM25 has uniform mesopores that facilitate mass transfer.

#### 3.6.4. Cyclic Bleaching Performance

**Figure 3:**
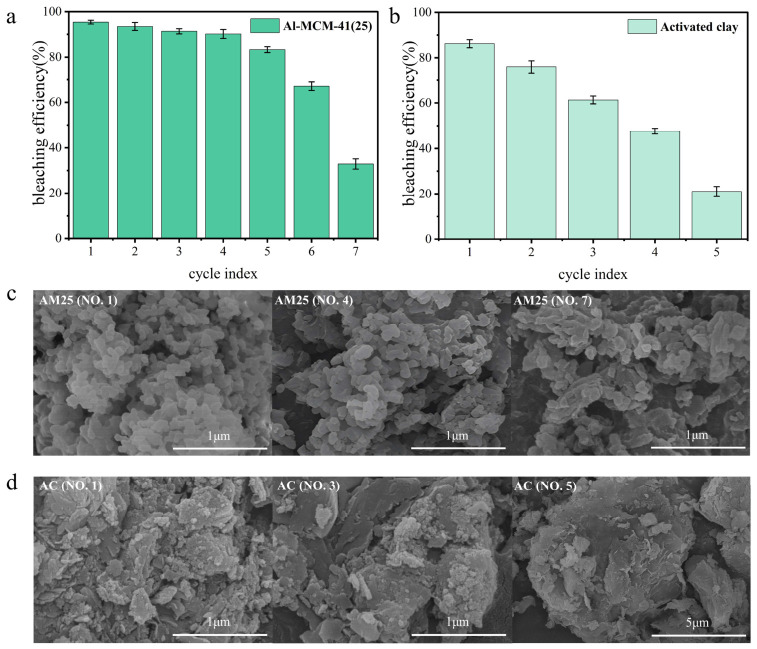
The cyclic bleaching performance of AM25 and AC for rapeseed oil. The cyclic bleaching efficiency of AM25 (**a**) and AC (**b**); SEM of AM25 before the 1st, 4th, and 7th adsorption cycles (**c**), and of AC before the 1st, 3rd, and 5th adsorption cycles (**d**). Note: AM25 refers to Al-MCM-41 with a Si/Al ratio of 25; AC stands for activated clay.

As shown in Figure 3a,b (70 °C, 20 min, 3 wt%), AM maintained >90% bleaching efficiency over four cycles and still achieved 83.31% in the fifth cycle. AC reached 86.2% only in the first run and dropped below 80% in the second cycle. SEM supported these trends; AM25 maintained the agglomerated particles and unobstructed channels observed prior to its first use until its fourth use, with significant agglomeration and pore blockage only becoming apparent by the seventh use (Figure 3c). In contrast, the layered structure of AC began to break down by the third use with noticeable clumping appearing, and the clumping became more pronounced by the fifth use (Figure 3d). Combined with the higher residual oil content of AC (0.16%) compared to that of AM25 (0.02%), this suggests that the residual oil covering the AC surface and interlayer channels was an important cause of the rapid decline in its bleaching performance. Overall, AM25 shows higher reusability than AC.

## 4. Conclusions

A series of MCM-41 adsorbents with varying Si/Al ratios was studied for removing chlorophyll and carotenoids (lutein, β-carotene) from rapeseed oil. Under identical conditions (70 °C, 20 min, 3 wt%, vacuum), AM25 showed the best comprehensive performance, achieving 94.57% chlorophyll removal, 92.84% lutein removal, and 84.09% β-carotene removal, while decreasing POV and AV by 76.59% and 32.30%, respectively, with high retention of tocopherols (93.89%), phytosterols (98.73%), and squalene (94.56%). After the bleaching of rapeseed oil with the MCM-41 adsorbents, among the FFA homologues, C18:3 exhibited the highest adsorption owing to its highest degree of unsaturation. The bleaching process was primarily governed by the appropriate surface acidity (Si–OH/Si–OH–Al) and the uniform 2–4 nm mesoporous size of the MCM-41 series adsorbents. Within the MCM-41 series, chlorophyll removal was more sensitive to acidic sites, whereas carotenoid removal mainly depended on the combined effects of mesopore confinement and stronger acid sites. The adsorption process of chlorophyll and carotenoids on the MCM-41 series adsorbents was mainly described by the pseudo-second-order kinetic model and the Freundlich isotherm model, and the adsorption of chlorophyll and carotenoids on the MCM-41 series adsorbents was identified as a spontaneous, endothermic, entropy-driven, heterogeneous multilayer process dominated by physical adsorption. Finally, regeneration experiments demonstrated that AM25 did not release Al during the bleaching process. Instead, it reduced the Al content in rapeseed oil to below the detection limit (0.1 mg/kg), while maintaining 83.31% bleaching efficiency after five regeneration cycles. This study provides a theoretical basis and application guidance for the vacuum bleaching of vegetable oils with MCM-41 adsorbents.

Nevertheless, several limitations should be noted. First, the selective bleaching performance of Al-MCM-41 was evaluated only in rapeseed oil under the present experimental conditions, and its applicability to other vegetable oils with different pigment compositions, FFA levels, peroxide contents, and micronutrient profiles remains to be further verified. In addition, the bleaching experiments were conducted under laboratory conditions, and the performance under atmospheric pressure or industrially relevant processing conditions has not yet been evaluated, which may influence mass transfer behavior and adsorption efficiency. Moreover, a direct comparison with other modern adsorbents was not included, because differences in raw materials, processing conditions, and evaluation criteria across the literature studies may lead to non-equivalent benchmarking results. Second, in the Si/Al-tuned Al-MCM-41 series, pore structure, surface area, total acidity, acid strength, and acid site accessibility changed simultaneously; therefore, the individual contribution of each structural or acidic parameter to adsorption selectivity could not be fully separated. In addition, although the introduction of Al was inferred from the acidity characterization results, more direct structural evidence is still needed. In particular, solid-state ^27^Al MAS NMR ICP-OES or ICP-MS would provide more definitive information to distinguish framework Al from extra-framework Al species and to further clarify their respective roles in adsorption behavior. Third, although AM25 showed better reusability than activated clay, its bleaching efficiency still declined after repeated cycles, suggesting that residual oil retention, pigment accumulation, or partial pore blockage may limit long-term regeneration. Future studies should focus on standardized comparisons among different adsorbents under unified conditions, independently controlled pore structure and acidity, evaluation under atmospheric or industrial processing conditions, more detailed characterization of Al coordination environments, and improved regeneration strategies for practical oil refining applications.

## Figures and Tables

**Table 1:** Pore parameters and NH_3_-TPD parameters for the MCM-41 series and AC adsorbents.

Sample	Peak	Peak Temperature (°C)	Acidity (mmol/g)	Total Acidity (mmol/g)	Average Pore Size (nm)	Surface Area (m^2^/g)	Pore Volume (cm^3^/g)
Activated clay	1	94.28	0.4175	0.8564	9.3	151	0.289
	2	576.26	0.4389				
Na-MCM-41	1	85.9	0.0082	0.0280	3.1	1048	0.740
	2	668.2	0.0198				
H-MCM-41	1	106.37	0.0940	0.2024	3.4	882	0.775
	2	476.04	0.1084				
Al-MCM-41(80)	1	104.95	0.2041	0.3557	4.0	813	0.890
	2	416.42	0.1516				
Al-MCM-41(60)	1	105.42	0.2642	0.4307	3.9	796	0.859
	2	467.11	0.1665				
Al-MCM-41(40)	1	114.76	0.3075	0.5035	3.9	791	0.848
	2	493.65	0.1960				
Al-MCM-41(25)	1	113.54	0.3408	0.5502	3.8	777	0.825
	2	496.62	0.2094				
Al-MCM-41(15)	1	118.01	0.3941	0.5561	3.7	698	0.704
	2	509.89	0.1619				

**Table 2:** Changes in the content of minor components in rapeseed oil after bleaching using the MCM-41 series and AC adsorbents.

Bleaching Oil	Tocopherol (mg/kg)				Phytosterol (mg/100 g)				Squalene (mg/100 g)	FFA (mg/100 g)				
	α-Tocopherol	γ-Tocopherol	δ-Tocopherol	Total Tocopherol	Brassicasterol	Campesterol	Sitosterol	Total Phytosterol		C18:0	C18:1	C18:2	C18:3	Total FFA
PRO	255.92 ± 7.29 ^a^	381.04 ± 5.57 ^a^	15.17 ± 0.10 ^a^	652.14 ± 11.93 ^a^	98.04 ± 0.63 ^a^	297.41 ± 4.66 ^a^	450.02 ± 7.25 ^a^	845.47 ± 11.93 ^a^	3.53 ± 0.16 ^a^	0.93 ± 0.03 ^b^	17.86 ± 0.09 ^a^	8.01 ± 0.05 ^a^	3.31 ± 0.04 ^a^	30.11 ± 0.04 ^a^
AC	213.42 ± 4.99 ^c^	340.96 ± 5.81 ^b^	13.09 ± 1.07 ^c^	567.47 ± 2.52 ^c^	93.41 ± 0.22 ^d^	287.19 ± 0.90 ^b^	431.98 ± 1.84 ^c^	812.59 ± 2.74 ^c^	3.22 ± 0.02 ^b^	1.11 ± 0.04 ^a^	18.38 ± 0.63 ^a^	8.44 ± 0.32 ^a^	3.52 ± 0.39 ^a^	31.45 ± 1.36 ^a^
NM	234.12 ± 13.9 ^b^	374.54 ± 4.24 ^a^	13.87 ± 0.13 ^bc^	622.53 ± 9.8 ^b^	93.82 ± 0.40 ^cd^	292.58 ± 1.11 ^a^	439.82 ± 2.50 ^b^	826.21 ± 3.71 ^b^	3.50 ± 0.13 ^a^	0.81 ± 0.07 ^cd^	16.53 ± 1.31 ^b^	7.44 ± 0.68 ^b^	2.9 ± 0.28 ^b^	27.68 ± 2.22 ^b^
HM	230.54 ± 2.84 ^b^	378.25 ± 3.4 ^a^	13.91 ± 0.14 ^b^	622.71 ± 5.55 ^b^	94.57 ± 0.45 ^bc^	295.27 ± 2.24 ^a^	444.82 ± 3.15 ^ab^	834.65 ± 3.14 ^b^	3.47 ± 0.14 ^a^	0.87 ± 0.05 ^bc^	13.87 ± 0.16 ^c^	6.39 ± 0.07 ^c^	2.56 ± 0.08 ^c^	23.69 ± 0.29 ^c^
AM80	226.45 ± 2.77 ^b^	375.17 ± 3.69 ^a^	13.88 ± 0.2 ^bc^	615.5 ± 3.43 ^b^	94.93 ± 0.85 ^b^	295.48 ± 3.79 ^a^	445.2 ± 1.71 ^ab^	835.60 ± 2.88 ^b^	3.47 ± 0.09 ^a^	0.86 ± 0.01 ^bc^	13.32 ± 0.11 ^cd^	6.22 ± 0.07 ^cd^	2.54 ± 0.18 ^c^	22.93 ± 0.32 ^cde^
AM60	225.08 ± 5.15 ^bc^	373.35 ± 3.91 ^a^	13.84 ± 0.13 ^bc^	612.26 ± 5.53 ^b^	94.77 ± 0.23 ^bc^	295.29 ± 3.53 ^a^	445.12 ± 2.46 ^ab^	835.19 ± 1.14 ^b^	3.42 ± 0.07 ^a^	0.86 ± 0.03 ^bc^	13.4 ± 0.14 ^cd^	6.17 ± 0.04 ^cde^	2.43 ± 0.04 ^c^	22.85 ± 0.12 ^cde^
AM40	223.8 ± 5.96 ^bc^	373.57 ± 4.32 ^a^	13.82 ± 0.4 ^bc^	611.19 ± 9.55 ^b^	94.75 ± 0.71 ^bc^	294.96 ± 1.86 ^a^	445.05 ± 0.49 ^ab^	834.75 ± 2.11 ^b^	3.42 ± 0.05 ^a^	0.78 ± 0.04 ^d^	12.49 ± 0.52 ^d^	5.75 ± 0.19 ^de^	2.31 ± 0.14 ^c^	21.33 ± 0.87 ^e^
AM25	224.86 ± 2.03 ^bc^	373.75 ± 1.77 ^a^	13.67 ± 0.34 ^bc^	612.28 ± 2.74 ^b^	94.70 ± 0.56 ^bc^	294.83 ± 1.63 ^a^	444.96 ± 2.30 ^ab^	834.49 ± 1.74 ^b^	3.40 ± 0.03 ^a^	0.78 ± 0.04 ^d^	12.59 ± 0.1 ^d^	5.7 ± 0.19 ^e^	2.4 ± 0.07 ^c^	21.46 ± 0.33 ^de^
AM15	226.92 ± 2.89 ^b^	375.54 ± 3.67 ^a^	13.82 ± 0.17 ^bc^	616.27 ± 4.89 ^b^	94.33 ± 0.59 ^bcd^	294.02 ± 1.79 ^a^	439.55 ± 3.46 ^b^	827.90 ± 5.81 ^b^	3.39 ± 0.07 ^a^	0.77 ± 0.04 ^d^	13.66 ± 0.15 ^c^	6.32 ± 0.05 ^c^	2.44 ± 0.08 ^c^	23.19 ± 0.06 ^cd^

Note: Different lowercase letters indicate significant differences following Duncan’s test, with *p* < 0.05. PRO denotes pre-bleaching rapeseed oil; NM denotes Na-MCM-41; HM denotes H-MCM-41; and AM80, AM60, AM40, AM25 and AM15 denote Al-MCM-41 with Si/Al ratios of 80, 60, 40, 25 and 15, respectively.

**Table 3:** The kinetic, isothermal and thermodynamic fitting parameters for the bleaching of rapeseed oil using MCM-41 series adsorbents and activated carbon.

Target Pigment	Adsorbents	Pseudo-First-Order Model				Pseudo-Second-Order Model				Langmuir				Freundlich			ΔH^0^ (kJ/mol)	ΔS^0^ (J/mol·K)	ΔG^0^ (kJ/mol)						R^2^
		*q_e_*	*k_1_*	*h_0_*	R^2^	*q_e_*	*k_2_*	*h_0_*	R^2^	*q_m_*	*K_L_*	*R_L_*	R^2^	*K_F_*	*n*	R^2^			50 °C	60 °C	70 °C	80 °C	90 °C	100 °C	
Chlorophyll	AC	0.336	3.185	1.070	0.9955	0.342	24.332	2.838	0.9993	2.127	0.258	0.263	0.983	0.426	1.358	0.994	35.83	154.08	−13.53	−15.31	−17.48	−18.59	−20.29	−21.29	0.978
	NM	0.191	0.914	0.174	0.9948	0.202	6.628	0.270	0.9910	0.483	0.143	0.392	0.959	0.080	1.777	0.949	14.83	73.42	−8.72	−9.81	−10.38	−11.10	−11.85	−12.46	0.976
	HM	0.262	1.305	0.342	0.9713	0.275	7.075	0.536	0.9958	0.564	0.483	0.160	0.935	0.198	2.326	0.986	18.32	89.94	−10.78	−11.44	−12.82	−13.27	−14.31	−15.26	0.969
	AM80	0.316	2.231	0.705	0.9584	0.327	12.170	1.300	0.9861	0.997	0.512	0.153	0.965	0.328	1.878	0.996	22.08	112.72	−14.16	−15.62	−16.58	−17.85	−18.76	−19.88	0.987
	AM60	0.333	3.047	1.016	0.9912	0.340	22.063	2.543	0.9987	1.836	0.368	0.200	0.970	0.476	1.453	0.980	21.92	114.39	−14.93	−16.24	−17.36	−18.53	−19.54	−20.70	0.996
	AM40	0.335	3.525	1.182	0.9970	0.340	29.999	3.466	0.9985	1.966	0.379	0.195	0.985	0.518	1.416	0.990	22.04	116.26	−15.40	−16.81	−17.90	−19.01	−20.03	−21.37	0.992
	AM25	0.340	4.003	1.362	0.9987	0.344	39.082	4.623	0.9996	2.114	0.387	0.192	0.988	0.567	1.385	0.995	24.24	123.54	−15.54	−17.03	−18.17	−19.40	−20.56	−21.81	0.995
	AM15	0.339	4.071	1.381	0.9989	0.343	42.128	4.947	0.9986	2.241	0.414	0.182	0.985	0.644	1.359	0.997	25.30	127.28	−15.82	−17.02	−18.31	−19.84	−20.80	−22.38	0.994
Carotenoid	AC	1.320	1.244	1.642	0.9572	1.393	1.281	2.486	0.9921	2.391	0.179	0.099	0.956	0.656	2.925	0.989	57.34	210.02	−11.23	−13.08	−14.44	−16.98	−18.27	−21.44	0.981
	NM	0.411	0.882	0.362	0.9688	0.438	2.836	0.544	0.9940	1.009	0.035	0.362	0.954	0.110	2.203	0.961	16.14	67.22	−5.62	−6.24	−6.78	−7.57	−8.44	−8.85	0.982
	HM	0.874	0.834	0.729	0.9606	0.927	1.356	1.166	0.9885	1.825	0.052	0.272	0.963	0.239	2.223	0.972	25.18	103.71	−8.39	−9.43	−10.03	−11.52	−12.68	−13.41	0.973
	AM80	1.143	2.585	2.954	0.9897	1.169	4.735	6.476	0.9989	2.081	0.097	0.168	0.920	0.410	2.538	0.979	33.37	131.91	−9.39	−10.63	−11.38	−13.20	−14.81	−15.80	0.972
	AM60	1.299	2.243	2.915	0.9752	1.339	3.113	5.583	0.9940	2.352	0.133	0.128	0.989	0.551	2.719	0.995	33.73	138.19	−10.70	−12.40	−13.74	−15.23	−16.52	−17.55	0.987
	AM40	1.413	2.041	2.883	0.9688	1.460	2.493	5.313	0.9935	2.559	0.185	0.096	0.972	0.692	2.853	0.995	34.49	144.42	−12.11	−13.58	−15.22	−16.49	−17.79	−19.43	0.996
	AM25	1.527	1.927	2.943	0.9628	1.582	2.111	5.280	0.9910	2.853	0.344	0.054	0.977	0.983	3.181	0.988	38.62	161.03	−13.19	−14.92	−17.13	−18.11	−20.08	−21.10	0.972
	AM15	1.501	1.954	2.932	0.9539	1.555	2.155	5.212	0.9859	2.678	0.321	0.058	0.974	0.929	3.278	0.981	36.39	153.44	−12.89	−14.69	−16.79	−17.91	−19.03	−20.73	0.968

Note: AC denotes activated clay; NM denotes Na-MCM-41; HM denotes H-MCM-41; and AM80, AM60, AM40, AM25 and AM15 denote Al-MCM-41 with Si/Al ratios of 80, 60, 40, 25 and 15, respectively.

**Table 4:** The effect of elution solvent on the reusability of AM25.

Adsorbent	Elution Solvent	Pigment Recovery After the First Bleaching Wash (%)			Second Bleaching Efficiency (%)	Oil Recovery (%)
		Chlorophyll	Lutein	β-Carotene		
AM25	n-Hexane	85.08 ± 3.86	89.78 ± 1.33	99.28 ± 1.65	81.04 ± 0.74	99.98 ± 0.07
	Ethanol	98.56 ± 2.53	98.17 ± 2.21	86.08 ± 4.4	87.42 ± 1.17	99.91 ± 0.16
	Ethanol: n-hexane = 5:1	99.4 ± 2.92	98.8 ± 1.87	99.24 ± 1.89	92.82 ± 2.33	99.98 ± 0.22
AC	Ethanol: n-hexane = 5:1	98.74 ± 1.49	98.58 ± 1.33	99.12 ± 1.03	75.86 ± 2.79	99.84 ± 0.29

Note: AM25 denotes Al-MCM-41 (Si/Al = 25); AC denotes activated clay.

## Data Availability

The original contributions presented in the study are included in the article/Supplementary Materials. Further inquiries can be directed to the corresponding author.

## Supplementary Materials

The following supporting information can be downloaded at https://www.mdpi.com/article/10.3390/foods15101738/s1, Supplementary Figure S1: Kinetic fitting curves for pseudo-first-order (PFO) and pseudo-second-order (PSO) adsorption of chlorophyll (a) and carotenoids (b) in rapeseed oil using y MCM-41 series and AC adsorbents; Supplementary Figure S2: Langmuir and Freundlich fitting curves for chlorophyll adsorption by MCM-41 series and AC adsorbents; Supplementary Figure S3: Langmuir and Freundlich fitting curves for carotenoid adsorption by MCM-41 series and AC adsorbents; Supplementary Figure S4: Thermodynamic fitting curves for bleaching of rapeseed oil chlorophyll (a) and carotenoids (b) using MCM-41 series and AC adsorbents.
