# Supplementary material for: Bleaching Performance and Mechanism of Al-MCM-41 Tuned by Si/Al in Rapeseed Oil
Source: Foods. 2026 May 14;15(10):1738. doi: 10.3390/foods15101738 (PMC13205917; doi:10.3390/foods15101738)
Supplement: Supplementary file 1 [file foods-15-01738-s001.zip › foods-4293342-supplementary.pdf]

Supplementary materials

Bleaching Performance and Mechanism of Al-MCM-41 Tuned by Si/Al in Rapeseed Oil

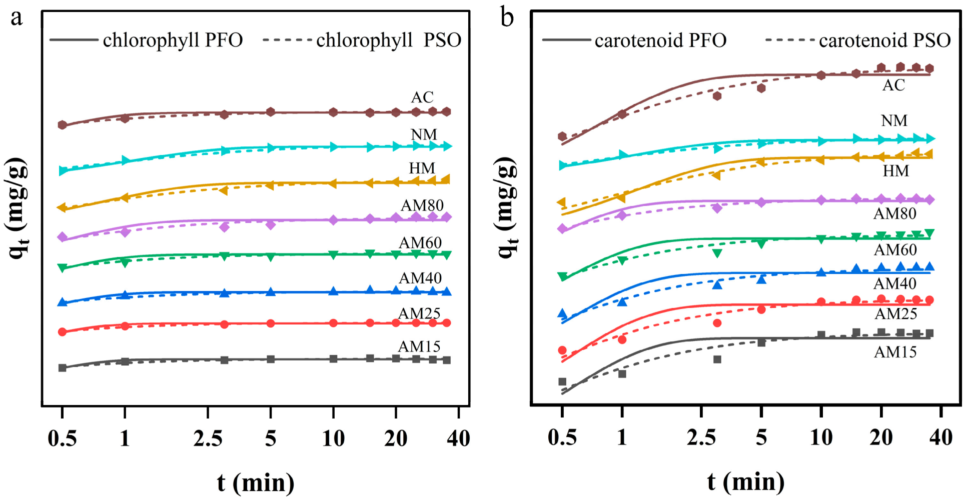

Supplementary Figure S1 Kinetic fitting curves for pseudo-first-order (PFO) and pseudo-second-order (PSO) adsorption of chlorophyll (a) and carotenoids (b) in rapeseed oil using y MCM-41 series and AC adsorbents.

Note: AC denotes activated clay; NM denotes Na-MCM-41; HM denotes H-MCM-41; AM80, AM60, AM40, AM25 and AM15 denote Al-MCM-41 with Si/Al ratios of 80, 60, 40, 25 and 15, respectively.

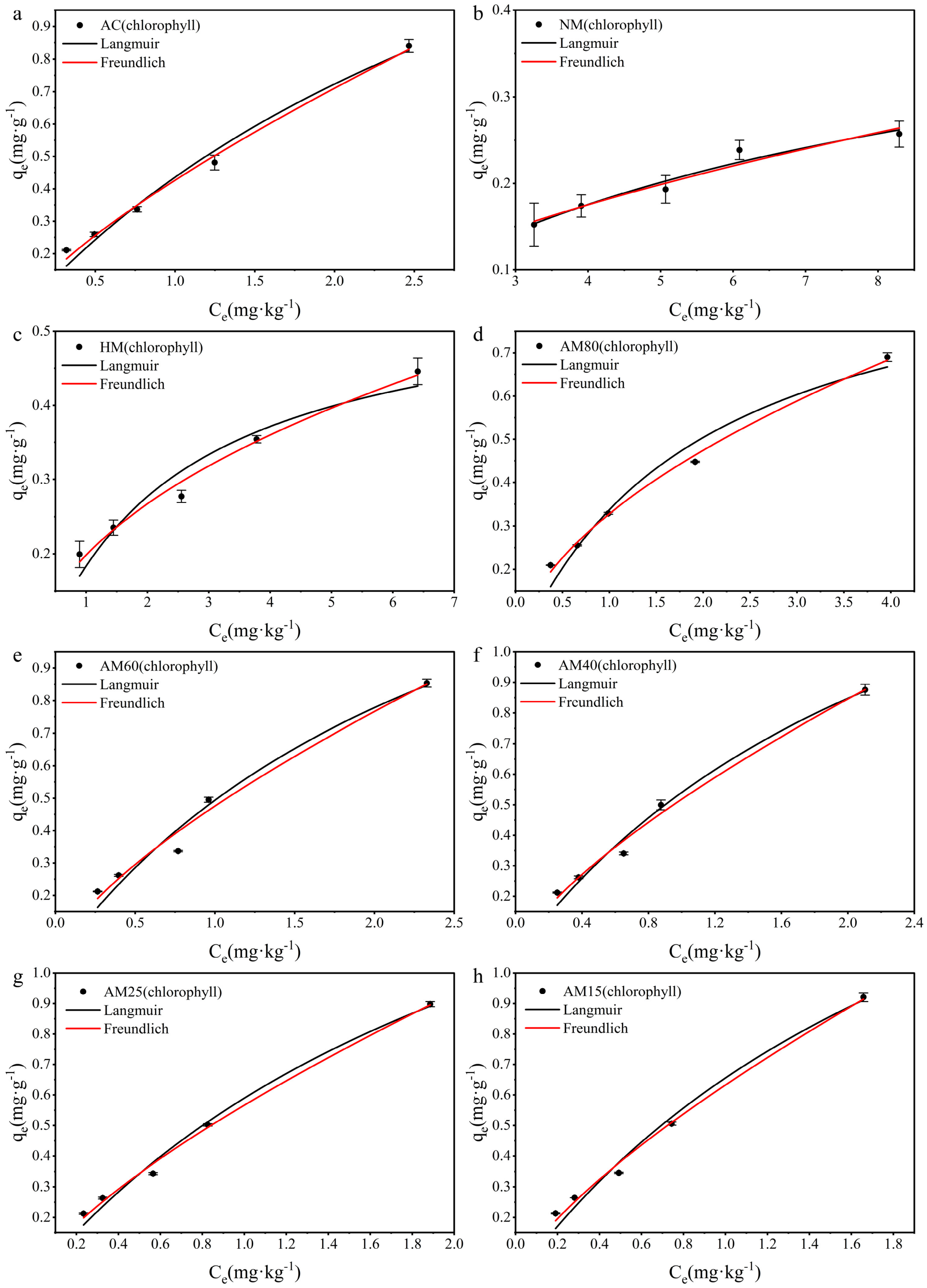

Supplementary Figure S2 Langmuir and Freundlich fitting curves for chlorophyll adsorption by MCM-41 series and AC adsorbents.

Note: AC denotes activated clay; NM denotes Na-MCM-41; HM denotes H-MCM-41; AM80, AM60, AM40, AM25 and AM15 denote Al-MCM-41 with Si/Al ratios of 80, 60, 40, 25 and 15, respectively.

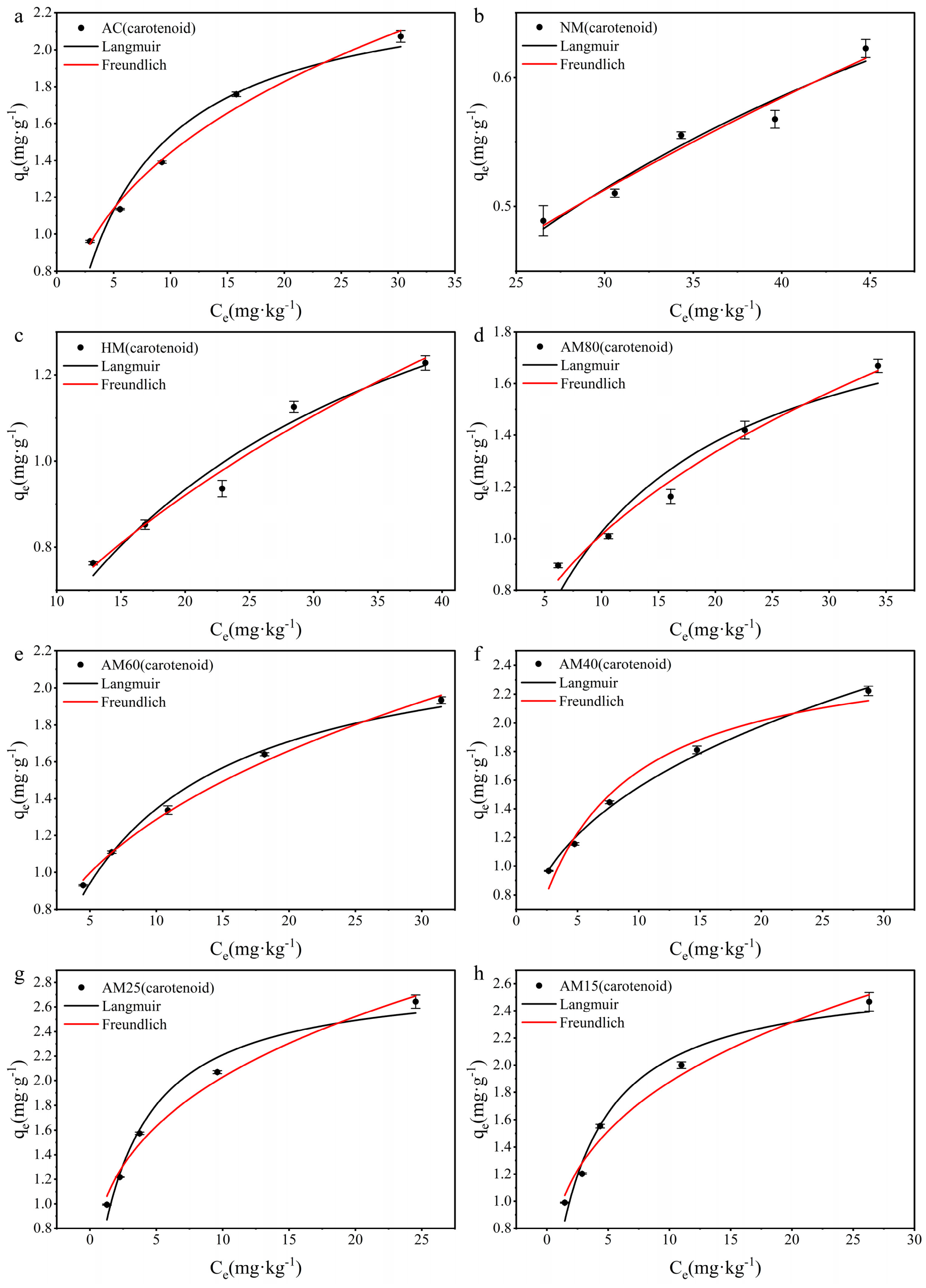

Supplementary Figure S3 Langmuir and Freundlich fitting curves for carotenoid adsorption by MCM-41 series and AC adsorbents.

Note: AC denotes activated clay; NM denotes Na-MCM-41; HM denotes H-MCM-41; AM80, AM60, AM40, AM25 and AM15 denote Al-MCM-41 with Si/Al ratios of 80, 60, 40, 25 and 15, respectively.

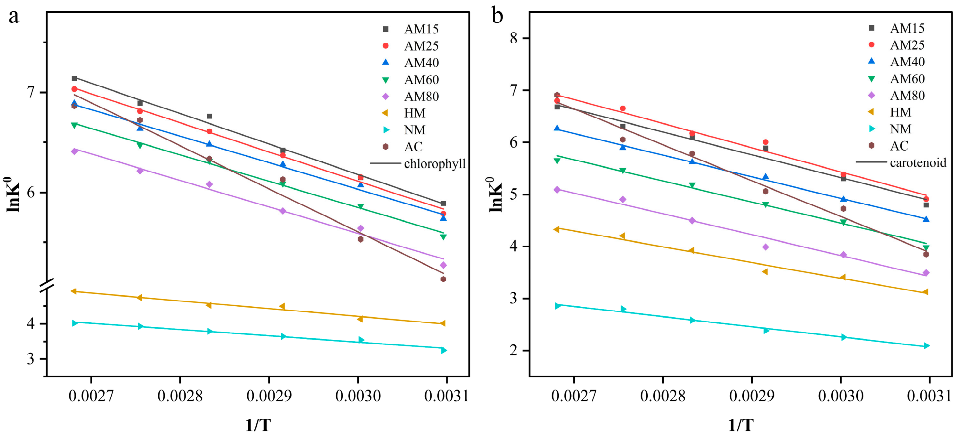

Supplementary Figure S4 Thermodynamic fitting curves for bleaching of rapeseed oil chlorophyll (a) and carotenoids (b) using MCM-41 series and AC adsorbents.

Note: AC denotes activated clay; NM denotes Na-MCM-41; HM denotes H-MCM-41; AM80, AM60, AM40, AM25 and AM15 denote Al-MCM-41 with Si/Al ratios of 80, 60, 40, 25 and 15, respectively.
